# Differentiating Self-Projection from Simulation during Mentalizing: Evidence from fMRI

**DOI:** 10.1371/journal.pone.0121405

**Published:** 2015-03-25

**Authors:** Matthias Schurz, Christoph Kogler, Thomas Scherndl, Martin Kronbichler, Anton Kühberger

**Affiliations:** 1 Centre for Cognitive Neuroscience, University of Salzburg, Salzburg, Austria; 2 Faculty of Psychology, University of Vienna, Vienna, Austria; 3 Neuroscience Institute, Christian Doppler Clinic, Paracelsus Medical University, Salzburg, Austria; CEA.DSV.I2BM.NeuroSpin, FRANCE

## Abstract

We asked participants to predict which of two colors a similar other (student) and a dissimilar other (retiree) likes better. We manipulated if color-pairs were two hues from the same color-category (e.g. green) or two conceptually different colors (e.g. green versus blue). In the former case, the mental state that has to be represented (i.e., the percept of two different hues of green) is predominantly non-conceptual or phenomenal in nature, which should promote mental simulation as a strategy for mentalizing. In the latter case, the mental state (i.e. the percept of green versus blue) can be captured in thought by concepts, which facilitates the use of theories for mentalizing. In line with the self-projection hypothesis, we found that cortical midline areas including vmPFC / orbitofrontal cortex and precuneus were preferentially activated for mentalizing about a similar other. However, activation was not affected by the nature of the color-difference, suggesting that self-projection subsumes simulation-like processes but is not limited to them. This indicates that self-projection is a universal strategy applied in different contexts—irrespective of the availability of theories for mentalizing. Along with midline activations linked to self-projection, we also observed activation in right lateral frontal and dorsal parietal areas showing a theory-like pattern. Taken together, this shows that mentalizing does not operate based on simulation *or* theory, but that both strategies are used concurrently to predict the choices of others.

## Introduction

To successfully predict behavior or decisions of another person, we must understand his or her feelings, thoughts, goals, desires and preferences. The discovery of how the brain enables us to infer mental states of others is a central field of social neuroscience research.

Traditionally, there are two different approaches in explaining what mentalizing is in cognitive terms. One view is that people refer to their own mental processes as a model to understand and predict others. This view is often called simulation theory [1,2], and similar ideas were formulated in theories of social projection [3] and egocentricity [4]. In its purest form, simulation theory suggests that there is no need of knowledge about other minds in mentalizing: we directly understand the mental life of others by internally replicating (“simulating”) it without any explicit reflection [5]. The competing approach postulates that we predict other people’s thoughts and actions by applying a set of rules, which consists of naïve knowledge about the mental domain. Thus, people possess a theory of mind which consists, for example, of a set of laws on how external stimuli become inner states (e.g., perceptions) and how some inner states (e.g., beliefs and desires) lead to behavior (e.g., choices). According to this approach, called theory-theory [6,7] people predict the actions of others by ascribing beliefs, desires and other mental states to them.

Neuroimaging studies showed that some brain areas engaged during mentalizing, in particular the ventral mPFC, overlap with brain areas activated during self-related thought. This was interpreted as reflecting an instance of self-projection i.e. that people use knowledge about their own thoughts, feelings, and preferences as a guide to others [8–10]. Moreover, it was sometimes taken as support for simulation-based accounts [8], as it was found that brain activation in the ventral mPFC was higher for mentalizing about similar compared to dissimilar others [8,11] and that the concordance between one’s own choices and the predicted choices for other people is stronger when activation in the ventral mPFC is high during prediction [10]. These findings support simulation theory which postulates that perceivers only use own mental processes as a model to predict others when these individuals are in some way perceived as similar to the self.

The reviewed findings speak for self-projection and simulation as important strategies for mentalizing. However, theoretical accounts about these two strategies are currently not clearly differentiated. In the present study, we clarify to which extent self-projection implies simulation. One feature that distinguishes simulation from other forms of mentalizing is that simulation can take place even if we don’t have a concept for what the other person has on her mind.

To make that clear, we consider the conception of mental states in the philosophy of mind, see e.g. [12]. Mental states such as for example, thoughts, beliefs and desires have “intentionality”, which means they are *about* something (e.g. “John wants to have a *glass of water*”). For mentalizing, one therefore needs to think about a mental state like “he wants to have”, and about the thing this mental state is about, like “… glass of water”. With respect to the latter, it is assumed that we can mentally represent the things in this world in two basic varieties [13]. On the one hand, our mental representations of things can be composed of concepts and not have phenomenal (“what it is like”) features. On the other hand, our mental states like sensations mainly (or only) have phenomenal features but no conceptual basis.

This distinction in our mental representations has an interesting implication for mentalizing. For simulation, we can use any kind of our own mental representations and project them to another person, as for example “John probably has the same mental state (or feeling) as I do—although I can’t really tell what it is…”. Any form of hypothetical thinking or theorizing, on the other hand, must be based on mental representations composed of concepts, and is hardly possible for purely phenomenal content.

With respect to this distinction between phenomenal versus conceptual mental states, the case of colors is of interest. Our sensory perception of colors is very rich and can detect most nuanced differences. Our mental representations of colors, however, do not directly reflect the physical features of colors, but are organized according to conceptual (i.e., linguistic) categories, like “blue, green, red ...” [14]. For example, it was found [15,16] that if two colors are from two different conceptual categories (e.g. “green” vs. “blue”), they are easier to distinguish and remember than two colors with the same physical distance but from one conceptual category (e.g. two widely different hues of green). This shows that our mind stores and represents colors not only according to their physical features, but also according to conceptual categories.

This feature of color-stimuli is used in the present study to differentiate between self-projection and simulation. We asked participants to predict which out of two colors another person would prefer. In one condition, the two colors were two hues of the same color-category (e.g., two hues of green). In this situation the thing that the other person’s mental state is about (i.e. the experience of two hues of green) is predominantly phenomenal and non-conceptual in nature. Therefore, we expected that simulation takes place in this condition—i.e. an internal replication of mental states without explicit reflection. In another condition, we showed participants two different primary colors. Here, the thing the other’s mental state is about (i.e., primary color green compared to primary color blue) can be captured in thought by concepts, which should facilitate the use of a theory for mentalizing.

By manipulating the nature of the color-difference in our stimuli, we aim at finding brain areas preferentially engaged in simulation. To see how this relates to self-projection, we additionally manipulated the target person for mentalizing by asking students either to rate their own preference, to predict the preference of a student (similar other), or of a retiree (dissimilar other). The expectation based on self-projection is overlapping activation for self and similar other, but less overlaps for self and dissimilar other. Based on previous research, we expect to find these overlaps mainly in cortical midline areas, such as vmPFC and precuneus [8,9,11] Taken that self-projection is linked to mental simulation, we would expect the following for our color-manipulation: Overlapping activations in midline areas for self and similar other in trials that show two hues of the same color-category, but not (or less so) in trials that show two conceptually different colors. Moreover, this activation difference (similar colors > different colors) should be found for similar other but not for dissimilar other (i.e., we expect an interaction between self-other similarity and color-factor).

## Methods

### Participants

The participants for the fMRI study were 24 (12 male) native German speakers. 2 participants were left handed, the rest of them were right handed. All participants were undergraduate students at the University of Salzburg (Austria). Mean age was 21.4 years; age-range was 19–27. All participants had normal or corrected-to-normal vision and reported no history of neurological or psychiatric disease. Participants gave a written informed consent and received course credit for participation. The study was approved by the ethics committee of the University of Salzburg (“Ethikkommission der Paris Lodron-Universität Salzburg”).

### Tasks and Stimuli

#### Preference-decision task

**Fig 1:**
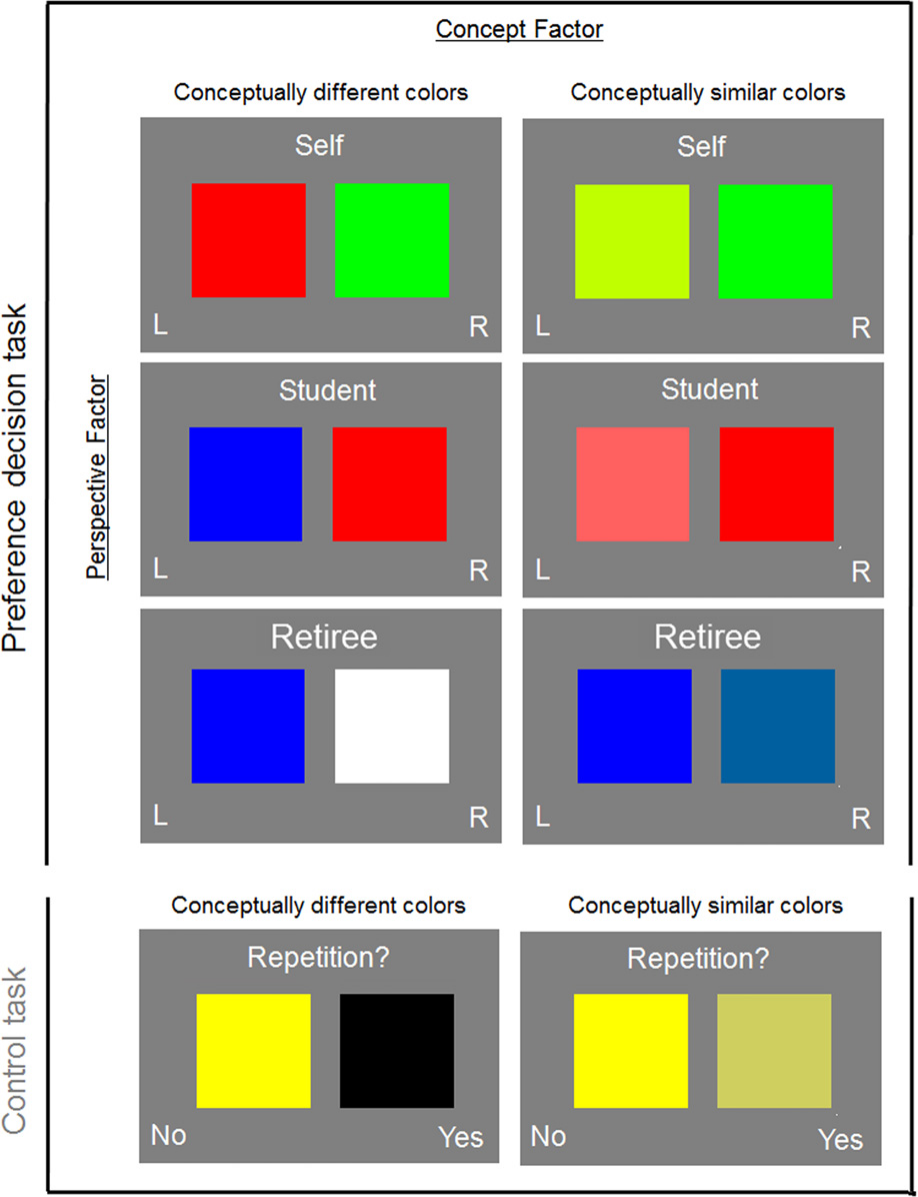
Task design. The preference decision task was arranged as a two-factorial design, with the factors perspective and concept. The perspective factor varied whether participants had to indicate their own preference (self), the preference of a student (similar other), or the preference of a retiree (dissimilar other) for one of the presented colors. The concept factor varied whether the two colors were conceptually similar or different. The control task asked for a one-back judgment on the color pairs.

In sum, the preference-decision task was arranged as a two-factorial design, with the factors perspective (self, student, retiree) and concept (different vs. conceptually similar colors). An illustration of the design and stimuli of our study is given in Fig. 1. Participants were told that their task would be to evaluate different colors in order to use them for the packaging of commercial products. In the *self* condition, participants had to choose for themselves which of two colors they prefer. In the *similar other* condition, they had to predict which of the two colors a typical student of the same gender would choose. Thus, the target person was similar to the participant with regard to gender, age and occupation. In the *dissimilar other* condition, participants had to predict the preferred color of a typical retiree of the opposite sex for each color pair (dissimilar with regard to gender, age and occupation). Participants responded with the index finger (“left color”) or the middle finger (“right color”) of their dominant hand. We presented pairs of clearly different primary colors in one half of the trials, and pairs of different hues of the same color-category in the other half of the trials.

#### One-back control task

The one-back task served as a control condition for the preference-decision task. Participants saw the same color-pairs as in the preference-decision task, but were asked for an old/new judgment on each pair (i.e., already seen on the trial immediately before). Participants responded with the index finger (“old”) or the middle finger (“new”).

#### Stimuli

Stimulus examples are shown in Fig. 1. For the conceptually different color-pairs, we arranged the primary colors (red, green, and blue) into pairs. The different primary colors in each of the resulting pairs had a minimum distance of 360 units in RGB space. Additional pairs of conceptually different colors were created with other primary / prototypical colors (black, white, yellow, cyan, and pink). Again, these additional color pairs were created in such a way that the two colors had a minimum distance of 360 units in RGB space. For the conceptually similar color-pairs, we created different hues of primary / prototypical colors. Different hues of a primary color were created by minimally varying its RGB configuration, with the constraint that there had to be a perceptual difference between the different hues—resulting pairs had a distance of 90 RGB units.

### Study design

In the fMRI experiment, the same sets of 10 conceptually similar and 10 conceptually different color pairs were presented in i) preference decision task for self, ii) preference decision task for a similar other iii) preference decision task for a dissimilar other and iv) the one-back task. The 20 color pairs were presented 4 times (at random positions) in the four tasks, resulting in a total of 320 trials.

The experiment was implemented as a block design. Blocks from the preference decision task, blocks from the one-back task and resting blocks were presented together in a pseudo-randomized order. A block showed 5 color-pairs for 3.2 seconds each, resulting in 16 seconds. During each block, an instruction cue was presented on top of the display (for the preference decisions: ‘self’, ‘student’ or ‘retiree’; for the one-back task: ‘old/new’). In addition, the two options for button-press were displayed on the corresponding sides of the screen (i.e., the option for the left response button was shown on the left side of the screen, the option for the right response button on the right side). Participants were instructed to respond as soon as they felt ready. There were a total of 320 trials, which were presented in 64 blocks, with 8 additional resting blocks which displayed the word ‘pause’ on the screen. The experiment was divided into two runs, which presented 36 blocks each. Blocks were separated by a short break of 3.2 seconds. Equal numbers of participants were assigned to one of three stimulus lists, each starting with a different condition (self, similar other or dissimilar other). Stimulus delivery and response registration were controlled by Presentation (Neurobehavioral Systems Inc., Albany, CA, USA). Before the experiments started, training sessions were used to familiarize participants with the tasks. The whole imaging experiment took approximately 35 minutes.

### fMRI data acquisition and analysis

Data were acquired on a 3T MRI scanner (Siemens Magnetom Trio, Siemens Medical Solutions, Erlangen, Germany) using a 32 channel head coil. Functional images were acquired were acquired with a T2* weighted echo-planar imaging (EPI) sequence (TR 2250 ms, TE 30 ms, Flip Angle 70°, matrix size 64 x 64, FOV 192 mm, in-plane resolution 3x3mm). Within one TR, 36 slices with a slice thickness of 3 mm were acquired. Functional imaging was divided into 2 scanning session, each collecting 350 images. Functional imaging took altogether 27 minutes. In addition, we collected a high-resolution structural image (1 x 1 x 1.2 mm) with a T1-weighted MPRAGE sequence, and a pair of fieldmaps (phase and magnitude images).

Data preprocessing and statistical analysis were performed with SPM 8 (http://www.fil.ion.ucl.ac.uk/spm) running in a MATLAB 7.6 environment (Mathworks Inc., Sherbon MA, USA). Functional images were realigned and unwarped using the parameters from the fieldmap images, and coregistered to the high-resolution structural image. The structural image was segmented into a grey matter, a white matter and a CSF image; then the three images were normalized to corresponding MNI T1 template images. The resulting parameters were used for spatial normalization of the functional images. Functional images were resampled to isotropic 3x3x3 mm voxels and smoothed with a 6 mm FWHM Gaussian Kernel.

Statistical analysis was implemented as a two stage random effects model. In the subject specific first level models, every condition from our experiment was modeled as a separate regressor within a general linear model. These regressors were convolved by a canonical hemodynamic response function. Six regressors coding for head-movements were modeled as covariates of no interest. Functional data in the first levels model were high pass filtered (cut-off 128 sec) and corrected for temporal autocorrelation by an AR(1) model [17]. At the first level, we calculated linear contrasts between parameter estimates, which reflect signal change for the different conditions versus passive rest.

For specific inference, we entered all eight images from each subject into a two-factorial repeated measures ANOVA (flexible factorial), with the factors task (self, student, retiree, one-back) and color (conceptually similar, conceptually dissimilar). Within this model, we computed planned *t*-contrasts. Results were thresholded at a voxelwise *p*<.001 and a cluster-level threshold of *p*<.05 FWE corrected.

## Results

### Behavioral Results

#### Internal consistency of choices

**Fig 2:**
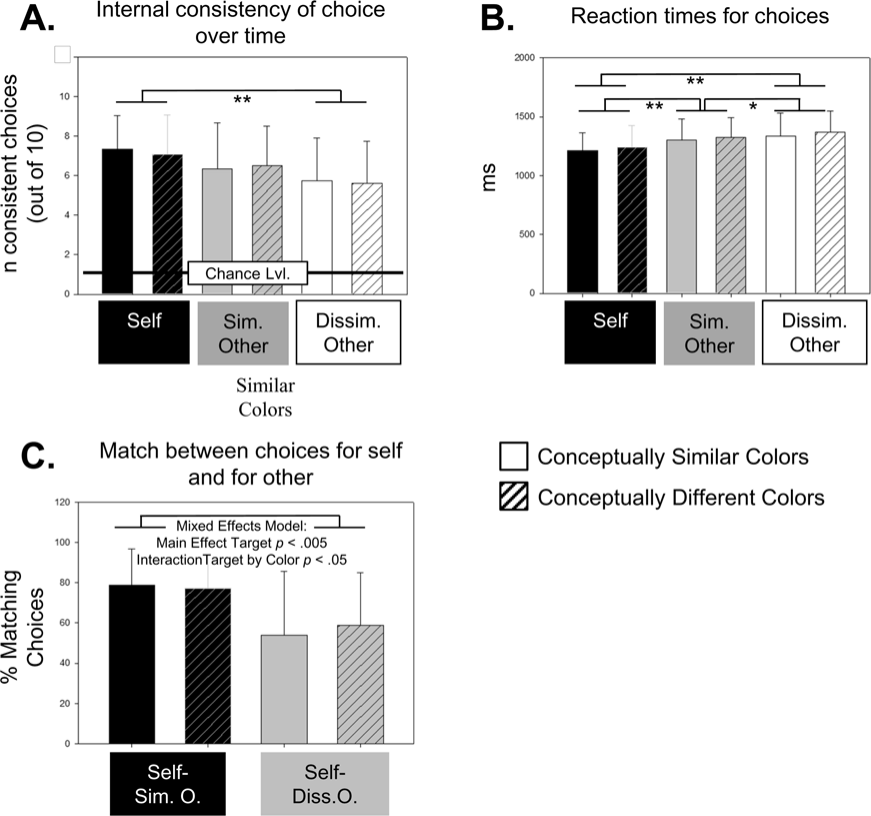
Behavioral Results. (A) Mean (SD) for internal consistency of choice over time. Gives the number of color-pairs (max = 10) where choices were consistent across all 4 repetitions throughout the experiment. (B) Mean (SD) reaction times for choices. (C) Mean (SD) percentage of matching choices for self and others, separately shown for self-similar other and self-dissimilar other. * *p*<.05 ** *p*<.01.

Because each color-pair was repeated for 4 times throughout our task, we could analyze how consistent participants were in their choices (for themselves and for others). We defined a choice as consistent if a participant selected the same color on all repetitions. The mean consistencies are shown in Fig. 2A. Participants were most consistent in their own choices (in about 7 out of 10 color-pairs). An ANOVA with the factors perspective (self, similar other, dissimilar other) and concept (different or similar colors) found a strong main effect of perspective on choice-consistencies, *F*(2,46) = 8.10, *p*<.001. Choices for self were more consistent than choices for for dissimilar others, post-hoc *t*-test *p*<.005. Choices for similar others only marginally differed from the other conditions. No other effects were significant in the ANOVA.

The chance-probability of selecting the same option on 4 consecutive trials of a two-alternative forced choice task is 1/8 (which equals to 1.25 out of 10 color-pairs, probability is determined by the binomial distribution). One sample *t*-tests showed that for all conditions, consistencies were highly significantly above chance level, *t*s(23) > 10.15, *p*s<.001. That means that all choices, irrespective of perspective and concept, were done in a systematic way by participants.

#### Reaction Times

Mean reaction times are shown in Fig. 2B. We performed repeated measures ANOVA on reaction times with factors perspective (self, similar other, dissimilar other) and concept (different or similar colors). We found a strong main effect of perspective (*F*(2,46) = 34.0, *p*<.001). The main effect of perspective showed that making predictions for a similar other and a dissimilar other took participants longer than making choices for self, post-hoc test *p*s<.001. Moreover, making predictions for a dissimilar other took participants longer than for a similar other (*p*<.05). No other effects of the ANOVA reached significance.

#### Match between choices for self and other

For each decision to be made in the experiment, we determined whether the choice for self was the same as for other. In other words, we created a categorical outcome variable that coded for each trial (i.e. each repetition of a color-pair), if the own-choice succeeded or failed to predict the choice made for other. To avoid possible confounds with the stimulus order, we only analyzed color-pairs where participants were consistent in their own choice across all 4 repetitions. That left us with 2734 out of 3792 trials for the analysis. We note that the total number of trials in the experiment would be 3840 (10 color-pairs x 4 repetitions x 2 color-conditions x 2 targets (self-similar other, self-dissimilar other) x 24 participants). However, due to an error in the stimulus coding, we could not analyze data from 1 out of 40 trials in the similar colors—similar other conditions. That left us with 3792 instead of 3840 trials. For an intuitive overview, the mean percentage of success-trials (where self- and other-choice was the same) is shown in Fig. 2C. We used the lme4 package [18] version 1.1–7 to estimate mixed effect models predicting the likelihood of matches between choices made for self and others. We started with a baseline model including participant, target (similar other, dissimilar other) and color (conceptually similar, conceptually dissimilar) as random effects. We then included target as a main effect in our model, which substantially improved the goodness of the model (*CHI*²(1) = 8.83, *p*<.01). In the next step, we included color as main effect and then the interaction between target and color. Color as a main effect did not improve the model (*CHI*²(1) = 1.29, *p* = .257), however including the interaction did so (*CHI*²(1) = 4.30, *p*<.05). As illustrated in Fig. 2C and reported in more detail in Table 1, participants had a significantly lower probability of having a match if the target was a dissimilar other (*b* = -1.71, *SE* = .28, *p*<.001, Odds Ratio *OR* = 0.18) compared to a dissimilar other. When looking at the interaction, we found that the target effect (reduced match for retiree) was more pronounced in the similar colors condition, *b* = 0.36, *SE* = 0.17, *p*<.05, *OR* = 1.44.

**Table 1:** Results of Linear Mixed Effects (LME) analysis of match between choices for self and other.

									OR		
	Df	AIC	BIC	logLik	ChiSq		b	z	M	LL	UL
Bl. Mod.	4	3233	3257	-1613							
+Target	5	3227	3256	-1608	8.83**						
+Color	6	3227	3263	-1608	1.29						
+Inter.	7	3225	3266	-1605	4.29*	FFX:					
						Intercept	3.27	6.88***	26.44	5.08	137.47
						Target	-1.71	-6.19***	0.18	0.09	0.37
						Color	-0.47	-1.64	0.62	0.22	1.75
						Inter.	0.36	2.07*	1.44	0.97	2.13

Bl. Mod… Baseline Model, Target… Varies if prediction was about similar versus dissimilar other, Color… Varies is prediction was made for similar versus dissimilar colors, Inter… Interaction between Target and Color, FFX… Fixed Effects, Df… Degrees of freedom, AIC Aikaike Information Criterion, BIC Bayesian Information Criterion, logLik… log Likelihood, ChiSq… Chi Square, b… non-standardized b coefficient, OR… Odd’s Ration, LL Lower Limit, UL… Upper Limit,

* *p*<.05,

** *p*<.01,

*** *p*<.001.

A possible caveat of our analysis of choice-matches is that we also found a difference in the internal consistency between self versus other-related choices. If choices are less consistent intrinsically for others compared to self, this could already explain the less than perfect match between self and other. To tackle the issue, we repeated our mixed effects model and only included color-pairs where participants were consistent on all 4 repetitions both for self and for other-choices (leaving 1767 out of 3792 trials for analysis). Results replicated what we found in our initial analysis, and the effects were even stronger in magnitude. Most importantly, the interaction between target and color again substantially improved the model (*CHI*²(1) = 13.76, *p*<.001). For space limitations, we report all other results of this analysis in S1 Table.

### Imaging Results

#### Activation for different color-pairs

**Fig 3:**
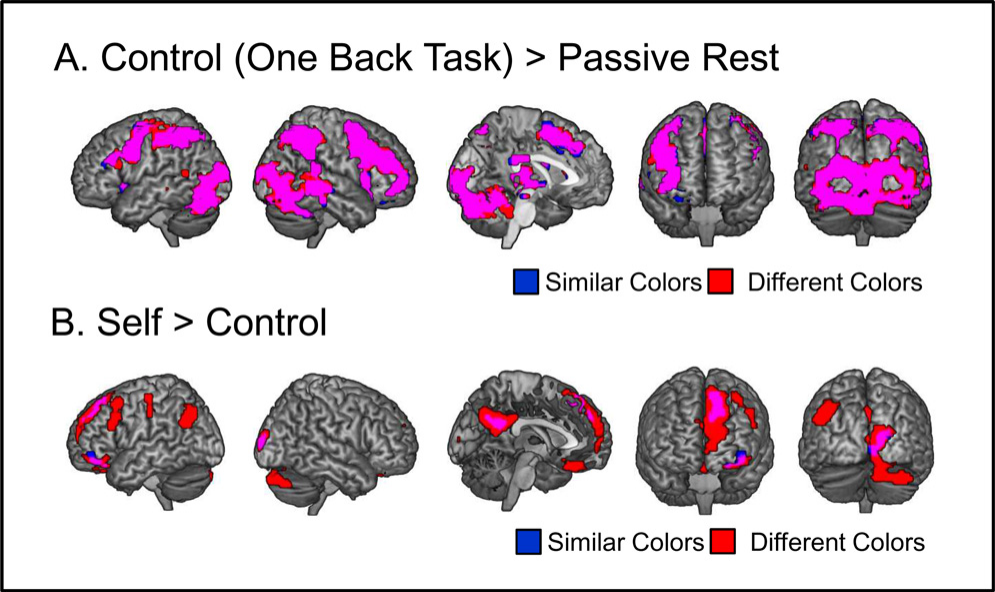
Baseline Contrasts. (A) Brain areas showing stronger activation for the one back task on color-pairs compared to passive rest (fixation cross viewing). (B) Brain areas showing stronger activation for self versus the one back (control) task. Results are separately shown for conceptually similar (blue) and conceptually different (red) colors. Overlapping areas are shown in purple. Voxel-level threshold of *p*<.001 and cluster extent *p*<.05 corrected.

We looked at the one-back control task to check for activation differences between conceptually similar and conceptually different color-stimuli. Fig. 3A shows the contrast one-back > passive rest separately for conceptually similar and conceptually different colors. Both contrasts show activation throughout the ventral visual processing stream and a typical fronto-parietal attention network, with frontal areas more pronounced on the right side. A direct comparison between color-pair conditions of the one-back task found no significant differences.

#### Self versus control (one-back task)

Our first goal was to map self-related processing. As passive rest is known to strongly activate the default mode network—which itself was linked to self-related cognition, e.g. [19]—we used the one-back task as control condition. Results from the contrast self > control were separately calculated for conceptually similar and conceptually different colors. Fig. 3B shows that for similar colors, self > control activated areas in the dorsal–posterior frontal cortex, posterior cingulate / precuneus, left lateral orbitofrontal areas, and areas in early visual cortex. For conceptually different colors, self > control activated the medial frontal cortex including dmPFC and vmPFC, in posterior cingulate / precuneus, left lateral orbitofrontal areas, the left temporo-parietal junction, right cerebellum, left middle frontal cortex, and left somato-sensory cortex. Anatomical details for results from contrasts self > controls are given in S2 Table.

#### Other versus self

**Fig 4:**
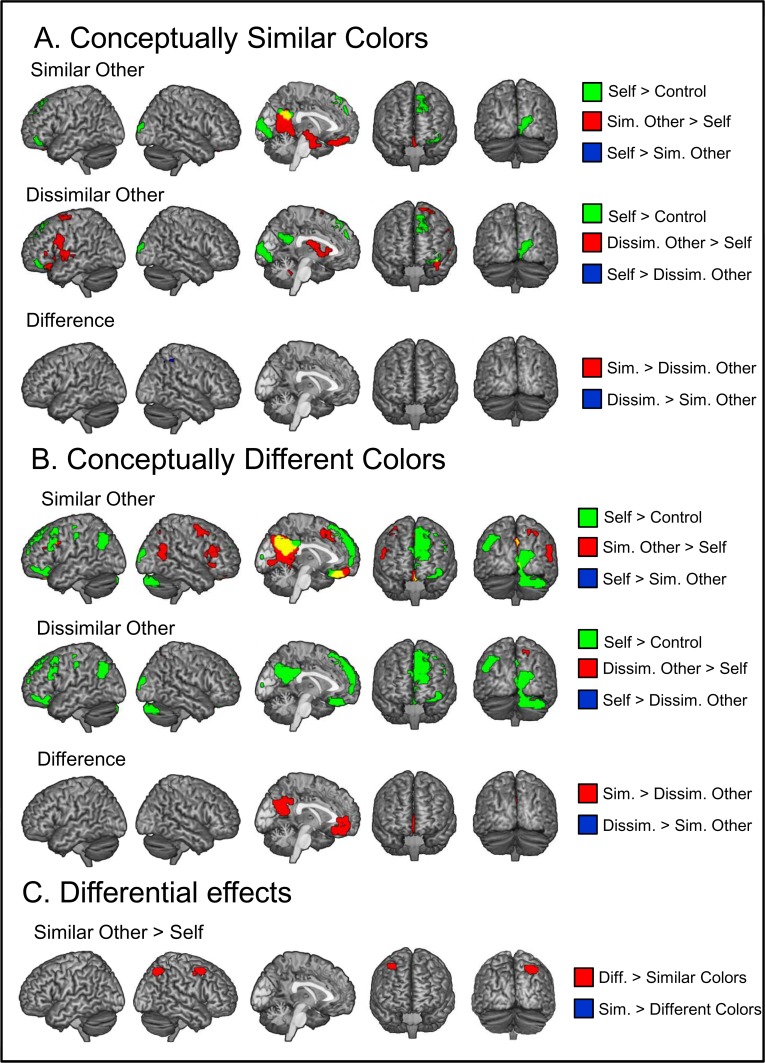
Differential Effects. (A) and (B): Green areas show higher activation for self versus control. Red and blue areas show differential activation with respect to the target of the preference judgment (self, similar other, dissimilar other). Results are separately shown for conceptually similar and dissimilar colors. (C). Red and blue areas show differences in differential activation with respect to target between conceptually different versus similar colors, i.e. an interaction between target and concept. Voxel-level threshold of *p*<.001 and cluster extent *p*<.05 corrected.

We compared brain activation for other versus self related judgments separately for conceptually similar and conceptually different colors. Results are shown in Fig. 4, and overlaid on the maps for the self > control contrast (described above). We also statistically tested for overlaps by calculating self > control (at voxel-wise *p*<.001 unc., cluster extent *p*<.05 corrected, and minimum extent 10 voxels) within a mask that only contained regions activated for other > self (determined by whole-brain analysis, voxel-wise *p*<.001 unc., cluster extent *p*<.05 corrected). Table 2 reports how many voxels of each other > self cluster were also activated for self > control (see column *Self>OneBack*). Using the same masking procedure, we also calculated how many voxels of each other > self cluster (e.g. in condition similar other) were also activated for the complementary other > self contrast (in condition dissimilar other). The results from this overlap analysis are given in the column *DissO>Self* or *SimO>Self*.

**Table 2:** Brain areas showing differential activation for mentalizing versus introspection.

	mni					vx overl.	vx overl.	vx overl.
Area	x	y	z	*z*	vx	with	with	with
SIMILAR COLORS								
						*Self >*	*DissO >*	*SimO >*
Similar Other > Self						*One Back*	*Self*	*DissO*
R precuneus	3	-55	19	5.40	410	43	-	33
L lingual	-12	-49	1	4.81	-			
L post. cingulate	-3	-49	28	3.81	-			
Orbitofrontal / vmPFC	0	41	-20	4.66	86	13	-	-
Orbitofrontal	0	32	-23	4.65	-			
R hippoc. / fusiform	39	-7	-26	4.53	76	-	13	-
R hippoc. / amygdala	18	-10	-11	3.80	-			
Self > Similar Other								
-	-	-	-	-	-	-	-	-
						*Self >*	*SimO >*	*DissO>*
Dissimilar Other > Self						*OneBack*	*Self*	*SimO*
L precentral	-57	11	31	4.54	143	-	-	25
L mid. temoral	-57	-16	1	3.95	-			
R thalamus	9	-7	19	4.34	465	-	12	-
R pallidum	18	8	-2	3.59	-			
L thalamus	-9	-7	19	3.86	-			
L putamen	-24	5	-2	4.07	-			
L IFG orbitalis / T.pole	-42	26	-17	4.10	-			
L sup. frontal	-21	2	67	4.21	91	-	-	-
L SMA	-12	8	70	3.57	-			
L cerebellum	-18	-58	-35	4.17	55	-	-	-
Self > Dissimilar Other								
-	-	-	-	-	-	-	-	-
Sim. Other > Diss. Other								
-	-	-	-	-	-	-	-	-
						*Self>*	*SimO>*	*DissO>*
Diss. Other > Sim. Other						*OneBack*	*Self*	*Self*
R inf. parietal	39	-46	49	3.69	51	-	-	10
R angular	27	-58	43	3.17	-			
DIFFERENT COLORS								
						*Self>*	*DissO>*	*SimO>*
Sim. Other > Self						*OneBack*	*Self*	*DissO*
L orbitofrontal	-3	44	-20	5.52	125	45	-	49
L vmPFC	-6	47	-17	5.08	-			
R precuneus	3	-52	22	5.03	1185	271	19	145
R post. cingulum	12	-46	22	4.84	-			
L post. cingulum	-6	-43	16	4.95	-			
L cuneus	-9	-61	25	4.53	-			
L precuneus	-3	-70	34	4.06	-			
R precentral	33	2	52	4.74	174	-	16*	-
R SMA	18	-4	49	4.12	-			
L IFG operc.	-36	8	22	4.51	152	-	36	-
L mid. frontal	-33	26	34	3.89	-			
L insula	-27	32	4	4.30	71	-	-	-
R sup. parietal	21	-64	55	4.31	56	-	20	-
R IFG triang.	39	26	25	4.05	66	-	-	-
L post. MTG	-60	-58	10	3.83	92	-	-	-
L TPJ /Angular	-57	-58	25	3.69	-			
L sup. frontal	-12	23	40	3.78	96	-	-	-
L SMA	-6	11	52	3.74	-			-
Self > Similar Other								
-	-	-	-	-	-	-	-	-
						*Self>*	*SimO>*	*DissO>*
Dissimilar Other > Self						*OneBack*	*Self*	*SimO*
R sup. parietal	18	-64	55	4.35	52	-	20	-
L precentral	-39	5	22	4.05	67	-	36	-
Self > Dissimilar Other								
-	-	-	-	-	-	-	-	-
						*Self>*	*DissO>*	*SimO>*
Sim. Other > Diss. Other						*OneBack*	*Self*	*Self*
L orbitofrontal	-3	32	-14	4.35	183	35	-	49
L vmPFC / ACC	-3	47	1	3.81	-			
L precuneus	-6	-61	31	3.69	169	93	-	145
L precuneus	-9	-52	16	3.56	-			
Diss. Other > Sim. Other								
-	-	-	-	-	-	-	-	-
INTERACTIONS								
SimO > Self: SC > DC								
-	-	-	-	-	-	-	-	-
						*Self>*	*DissO>*	*SimO>*
						*OneBack*	*Self*	*Self*
SimO > Self: DC > SC						SC / DC	SC / DC	SC / DC
R superior parietal	30	-64	52	4.31	77	- / -	- / -	- / 28
R mid. frontal	33	14	52	4.13	83	- / -	- / 11*	- / 69
R precentral	36	-4	46	4.01	-			
DissO > Self: SC > DC								
-	-	-	-	-	-	-	-	-
DissO > Self: DC > SC								
-	-	-	-	-	-	-	-	-
SimO vs DissO: SC vs DC^1^								
-	-	-	-	-	-	-	-	-

* Not significant at *p*<.05 cluster-level corrected.

^1^ The label “vs” subsumes directed contrasts in both directions, summarized here for brevity.

Abbreviations: SimO… Similar Other; DissO… Dissimilar Other, SC… Similar Colors, DC… Different Colors.

Results are separately shown for conceptually similar and conceptually different colors. Voxel-level threshold of *p*<.001 and cluster extent *p*<.05 corrected.

#### Conceptually Similar Colors

As shown Fig. 4A, we found higher activation for similar other > self in the ventral mPFC / orbitofrontal cortex, precuneus, and in right-hemispheric limbic areas including hippocampus and amygdala. Of these areas, only the precuneus and the orbitofrontal cortex / vmPFC were also significantly engaged during self > control (see Table 2, *Self>OneBack*). Moreover, among the areas activated for similar other > self, the right hippocampus / amygdala cluster was also activated for the contrast dissimilar other > self. No areas showed higher activation for self > similar other.

Next, we looked for areas with the contrast dissimilar other > self. We found activation in the left precentral gyrus, a large bilateral subcortical cluster including parts of thalamus, globus pallidus and putamen, in an area in the left superior frontal cortex / SMA, and in the left cerebellum (see Fig. 4A). None of these areas activated for dissimilar other > self was also activated during self > control, and only the right thalamus cluster was also activated for similar other > self. The reverse contrast looking for self > dissimilar other again found no significant results.

#### Conceptually Different Colors

As shown in Fig. 4B, higher activation for similar other > self was found in a large area in the precuneus and posterior cingulate cortex. Moreover, higher activation was found in the vmPFC / orbitofrontal cortex, left and right lateral frontal areas, and in the right posterior middle temporal gyrus and temporo-parietal junction. Further clusters showing stronger activation were found in right superior parietal lobule, right inferior frontal gyrus, and left superior frontal gyrus / SMA. As before, the precuneus and the orbitofrontal cluster showed overlaps with activation found for self > control. Overlapping areas for the contrasts similar other > self and dissimilar other > self were found in left frontal cortex and right superior parietal lobule, and for the right frontal cortex and precuneus. The reverse contrast self > similar other again showed no significant activation.

Higher activation for dissimilar other > self was found in the right superior parietal lobule and in the left precentral gyrus. Both areas were not activated for self > control. Moreover, we found that both areas were also activated by the contrast similar other > self. The reverse contrast self > dissimilar other again found no activation differences.

### Differences between mentalizing for similar and dissimilar others

We wanted to evaluate which brain areas were not only stronger activated for other > self, but also for similar other versus dissimilar other. Results are listed in Table 2 in the column *SimO>DissO* and accordingly *DissO>SimO*. Additionally, we also looked for differences in mentalizing about a similar versus a dissimilar other on the whole brain level. Results are listed in separate paragraphs at the end of Table 2.

#### Conceptually Similar Colors

We found that among the areas showing stronger activation for similar other > self, stronger activation for similar other > dissimilar other was found in the precuneus—see the leftmost column *SimO > DissO* in Table 2. We also looked at the contrast similar other > dissimilar other at the whole brain level. No additional areas were found. Among the areas that showed stronger activation for dissimilar other > self, only the left precentral gyrus area also showed stronger activation for dissimilar other > similar other. On the whole brain level, we additionally found an area in the right inferior parietal lobule, located medially and possibly in the intraparietal sulcus for dissimilar other > similar other.

#### Conceptually Different Colors

Among the areas that showed stronger activation for similar other > self, we found that the precuneus and the left vmPFC also showed stronger activation for similar other > dissimilar other. These two findings were also the only activation differences identified by our whole-brain search for similar other > dissimilar other. Among the areas showing stronger activation for mentalizing about a dissimilar other > self, none also showed stronger activation for dissimilar other > similar other.

### Differential patterns for conceptually similar versus different colors

All differential effects (e.g. similar other > self) were finally contrasted between conceptually similar and different colors. In other words, we looked for ‘target person’ by ‘color concept’ interactions. We used at voxel-wise *p*<.001 uncorrected together with cluster extent *p*<.05 corrected. Results are shown in the section *Interactions* in Table 2. For the contrast similar other > self, greater activation was found for conceptually different colors compared to similar colors in the right superior parietal lobe and in the right middle frontal gyrus. Activations are also shown in Fig. 4C. No other interactions showed significant effects.

## Discussion

In the present study participants predicted which of two colors a similar other (student) and a dissimilar other (retiree) would prefer. The novelty of our study is that we manipulated if color-pairs were two hues from the same color-category or two conceptually different colors (e.g. red versus blue). The former case should promote simulation, whereas the latter should facilitate theorizing as strategy for mentalizing. Based on this separation between simulation and theorizing, we searched for brain areas that support self-projection, i.e. overlaps in activation for predicting other’s preferences and making self-related judgments. This allowed us to evaluate if self-projection is linked to simulation or the use of theories.

On the behavioral level, we found an interaction between self-other similarity and the conceptual nature of colors. The interaction showed that, if colors were two hues from the same category (e.g. green), predictions made for a similar other were most closely corresponding to one’s own choice (almost 80% match), whereas predictions for a dissimilar other were most different from the own choice (around 50% match). This behavioral pattern speaks for simulation: The own choice is replicated if this is appropriate because of high self-other similarity and the absence of a conceptual basis that could promote theory use.

**Fig 5:**
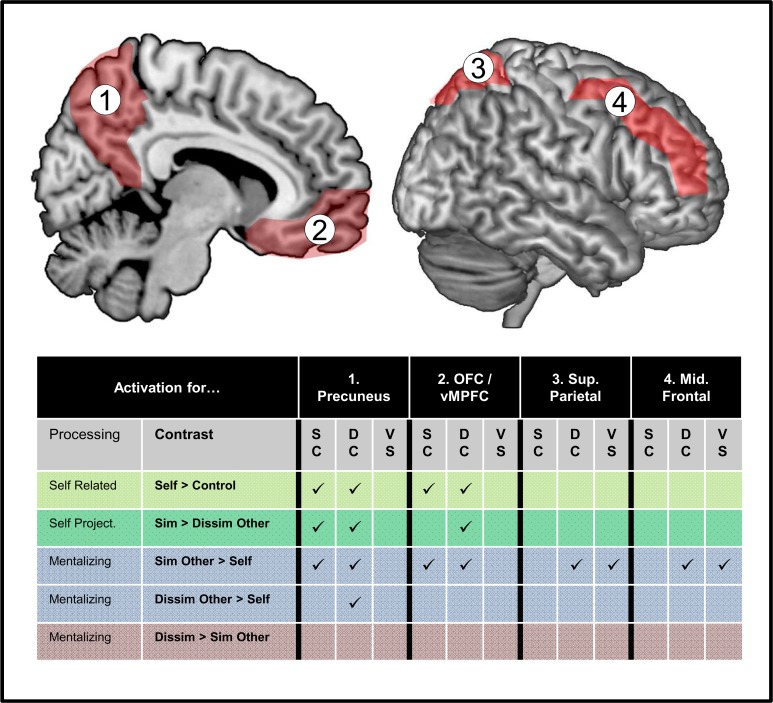
Summary of Main Findings. Activation was determined by whole brain analyses using different contrasts tapping into different cognitive processes (self-related cognition, self-projection, mentalizing). A “✓”symbol indicate that a contrast found activation at *p*<.001, *p*<.05 corrected in parts of that brain area. Detailed results are reported in Table 2. Contrast results are listed separately for conceptually similar colors (‘SC’) and conceptually different colors (‘DC’) and for the direct comparison between SC versus DC—labeled ‘VS’.

Brain activation findings are summarized in Fig. 5. As for the behavioral data, we found a clear main effect of target. The orbitofrontal cortex / vmPFC and the precuneus showed activation patterns in support of self-projection (self>control, similar other > self, similar other > dissimilar other). However, the pattern did not significantly differ between the condition promoting simulation and the condition promoting theorizing (i.e. equal for conceptually similar and conceptually different colors, respectively). Another main finding was that for right superior parietal and middle frontal gyrus, we found an interaction between self-projection and color-concept. That is, activation for similar-other > self was specifically found for conceptually dissimilar colors in those areas. The finding did not show a pattern in support of self-projection, as areas were not activated for self>control. The direct comparison between targets showed that this activation pattern (i.e. stronger for conceptually dissimilar versus similar colors) was not different for similar versus dissimilar other. These findings suggest that right parietal lobe was engaged more strongly for conceptually different colors, in line with theory. Interestingly, this process did not differ in strength for similar and dissimilar other.

### Midline areas

Our results replicate the standard findings related to self-projection [8,11,20,21]. Effects were found in the vmPFC and the precuneus, two structures strongly linked to self-related processing [22,23] and mentalizing [24–27]. In addition, we identified the orbitofrontal cortex, which is known to be frequently co-activated with the vmPFC [28] and important for computing the current value or desirability of an object or an action for the self [29,30]. In the present experiment, participants had to rate the pleasantness of one color in relation to a particular other color, which implies a judgment about the current rather than the absolute value of that color.

The activation patterns for vmPFC / orbitofrontal cortex and the precuneus are summarized in Fig. 5. In support of self-projection, we found activation differences for self>control, similar other > self, and similar > dissimilar other. The novel aspect of our study is that we additionally manipulated the conceptual basis for mentalizing. A central feature of a brain area that subserves simulation is that its workings do not require a concept for the thing that the other person’s mental state is about. For simulation, it is sufficient to simply (re-) experience a mental state of purely phenomenal or non-conceptual nature. Therefore mentalizing about conceptually similar colors should strongly engage brain areas subserving simulation. However, our results show that activation in cortical midline areas is not selective for conceptually similar colors, but also found for conceptually different colors. That is, we found the typical self-projection pattern in the precuneus and the ventral mPFC both for conceptually similar and conceptually different colors.

The absence of significant effects for our color-manipulation are surprising and do not support our working hypothesis that self-projection is linked to simulation. Rather, they suggest that self-projection is a strategy applied in different contexts—irrespective of the availability of theories for mentalizing.

### Right frontal and parietal areas

The other main finding of our study regards the right middle frontal gyrus and right superior parietal lobule. The activation patterns are illustrated in Fig. 5. Both areas were only activated by the mentalizing contrasts similar other > self. Moreover, we found that activation in these areas was only found for conceptually different colors, and not for conceptually similar colors. This difference survived a direct comparison.

The parietal cortex is engaged in making comparative judgments of stimuli on a particular dimension, like for example number, size and luminance [31]. It is also engaged in comparing and grouping stimuli according to color [32], and in the formation of color-concepts according to color-groupings [33]. The right middle frontal gyrus is specifically engaged when colors from different categories are presented relative to colors from the same category—irrespective of the size of the hue difference [34]. This indicates that the right middle frontal gyrus is representing or coding colors in categories.

These findings on the right parietal and middle frontal gyrus suggest that participants were categorizing the color-stimuli presented in our task more strongly when they had to predict the choice for another person compared to themselves. Categorizing colors allows using conceptual knowledge about these categories, which speaks for theorizing rather than simulation. It is of interest that this activation pattern speaking for theorizing was similarly strong for similar other and dissimilar other. This lets us conclude that mentalizing can include theory-like processes irrespective to the extent of self-projection or simulation.

### Hippocampus

Another observation was the implication of hippocampus and amygdala in predicting other’s choices. We found activation for the contrasts similar other > self and dissimilar other > self, but, interestingly, no activation for our self-projection contrasts. Activation did not significantly differ for the color-concept factor. For the hippocampus, one interpretation of the observed activation pattern could be that participants used the memory of their own choices to produce choices for others. In addition, activation in amygdala could reflect a re-instantiation of implicit knowledge linked to emotional value of the stimuli, see e.g., [35].

### Limitations

Our color-manipulation promotes different strategies for mentalizing (similar colors—simulation; different colors—theorizing), but does not guarantee a perfect separation. For example, one can argue that two hues of the same color can also be represented conceptually and linguistically (e.g. “lawn-green” versus “olive-green”). Nevertheless, evidence suggests that there is a measureable difference in processing of conceptually similar versus different color pairs. It was found that there are a few major color-categories, i.e. terms spontaneously used by participants for naming colors across the entire spectrum. In English, these are “red, pink, purple, blue, green, brown, yellow, orange” [36], see also [14]. Further research [15,16] showed that if two colors are from two different conceptual categories (e.g. “green” vs. “blue”), they are easier to distinguish and remember than two colors with the same physical distance but from one conceptual category (e.g. two widely different hues of green). Note that these differences were found notwithstanding the fact that the latter two could be described as e.g. “lawn-green” vs. “olive-green”. Thus, hues from different (compared to similar) categories have mental representations that differ more strongly in their conceptual nature. Brain activation differences found in our study can be linked to this relative difference; however, they do not reflect an all-or-none manipulation of simulation versus theorizing.

## Conclusion

We asked participants to predict which of two colors a similar other (student) and a dissimilar other (retiree) would prefer. During these judgments, we manipulated whether color-pairs were two colors from the same hue or two conceptually different colors (e.g. green versus blue). The former setting should promote simulation, whereas the latter should facilitate theorizing as strategy for mentalizing. We found two main effects: First we found a self-projection pattern in cortical midline areas—however, this was not affected by the conceptual nature of the color-difference. This suggests that self-projection subsumes simulation-like processes but may not be limited to them. Self-projection could be a universal strategy applied in different contexts—irrespective of the availability of theories for mentalizing. Second, we found a theory-like activation pattern in dorsal fronto-parietal areas—and this was not affected by the similarity of the target person. Put together, results show that mentalizing does not operate based on simulation *or* theory-theory, but that both strategies can be used concurrently to predict the choices of others, which is in line with hybrid-views, see e.g. [37].

## Supporting Information

S1 Table Results of Linear Mixed Effects (LME) analysis of match between choices for self and other—only trials with perfect internal consistency for self and other choices. (DOCX)

S2 Table Results for self > control task. Results are separately shown for conceptually similar and conceptually different colors. Voxel-level threshold of *p*<.001 and cluster extent *p*<.05 corrected.
(DOCX)

## References

[1] Gordon RM. Folk psychology as simulation. Mind Lang. 1986;1(2): 158–171.

[2] Heal J. Simulation, theory, and content In: Carruthers P, Smith PK, editors. Theories of theories of mind. Cambridge: Cambridge University Press; 1996 pp. 75–89.

[3] Ames DR, Iyengar SS. Appraising the unusual: Framing effects and moderators of uniqueness-seeking and social projection. J Exp Soc Psychol. 2005;41(3): 271–282.

[4] Dunning D, Hayes AF. Evidence for egocentric comparison in social judgment. J Pers Soc Psychol.1996;71(2): 213.

[5] Gallese V, Keysers C, Rizzolatti G. A unifying view of the basis of social cognition. Trends Cogn Sci. 2004;8(9): 396–403. 15350240 10.1016/j.tics.2004.07.002

[6] Churchland PM. Eliminative materialism and the propositional attitudes. J Philos. 1981;78: 67–90.

[7] Fodor JA. Psychosemantics: The problem of meaning in the philosophy of mind. Cambridge, MA: The MIT Press; 1987.

[8] Mitchell JP, Banaji MR, MacRae CN. The link between social cognition and self-referential thought in the medial prefrontal cortex. J Cogn Neurosci. 2005;17(8): 1306–1315. 16197685 10.1162/0898929055002418

[9] Mitchell JP. Inferences about mental states. Philos Trans R Soc Lond B Biol Sci. 2009;364(1521): 1309–1316. 10.1098/rstb.2008.0318 19528012 PMC2666715

[10] Tamir DI, Mitchell JP. Neural correlates of anchoring-and-adjustment during mentalizing. Proc Natl Acad Sci U S A. 2010;107(24): 10827–10832. 10.1073/pnas.1003242107 20534459 PMC2890763

[11] Mitchell JP, Macrae CN, Banaji MR. Dissociable medial prefrontal contributions to judgments of similar and dissimilar others. Neuron. 2006;50(4): 655–663. 16701214 10.1016/j.neuron.2006.03.040

[12] Pitt D. Mental Representation. In: Zalta EN, editor. The Stanford Encyclopedia of Philosophy. 2013. Available: http://plato.stanford.edu/archives/fall2013/entries/mental-representation. Accessed 14 February 2014.

[13] Boghossian PA. Content In: Kim J, Sosa E, editors. A Companion to Metaphysics. Oxford: Blackwell Publishers Ltd; 1995 pp. 94–96.

[14] Davidoff JB. Language and perceptual categorisation. Trends Cogn Sci. 2001;5(9): 382–387. 11520702 10.1016/s1364-6613(00)01726-5

[15] Kay P, Kempton W. What is the Sapir-Whorf hypothesis? Am Anthropol. 1984;86: 65–78.

[16] Roberson D, Davies I, Davidoff J. Color categories are not universal: replications and new evidence from a stone-age culture. J Exp Psychol Gen. 2000;129: 369–398. 11006906 10.1037//0096-3445.129.3.369

[17] Friston KJ, Glaser DE, Henson RN, Kiebel S, Phillips C, Ashburner J. Classical and Bayesian inference in neuroimaging: applications. Neuroimage. 2002;16(2): 484–512. 12030833 10.1006/nimg.2002.1091

[18] Bates D, Maechler M, Bolker BM, Walker S. lme4: Linear mixed-effects models using Eigen and S4; 2014. Preprint. Available: http://arxiv.org/abs/1406.5823. Accessed 20 December 2014.

[19] Spreng RN, Mar RA, Kim AS. The common neural basis of autobiographical memory, prospection, navigation, theory of mind, and the default mode: a quantitative meta-analysis. J Cogn Neurosci. 2009;21(3): 489–510. 10.1162/jocn.2008.21029 18510452

[20] Jenkins AC, Macrae CN, Mitchell JP. Repetition suppression of ventromedial prefrontal activity during judgments of self and others. Proc Natl Acad Sci U S A. 2008;105(11): 4507–4512. 10.1073/pnas.0708785105 18347338 PMC2393803

[21] Murray RJ, Schaer M, Debbané M. Degrees of separation: A quantitative neuroimaging meta-analysis investigating self-specificity and shared neural activation between self- and other-reflection. Neurosci Biobehav Rev. 2012;36: 1043–1059. 10.1016/j.neubiorev.2011.12.013 22230705

[22] van der Meer L, Costafreda S, Aleman A, David AS. Self-reflection and the brain: a theoretical review and meta-analysis of neuroimaging studies with implications for schizophrenia. Neurosci Biobehav Rev. 2010;34(6): 935–946. 10.1016/j.neubiorev.2009.12.004 20015455

[23] Northoff G, Heinzel A, de Greck M, Bermpohl F, Dobrowolny H, Panksepp J. Self-referential processing in our brain—a meta-analysis of imaging studies on the self. Neuroimage. 2006;31(1): 440–457. 16466680 10.1016/j.neuroimage.2005.12.002

[24] Bzdok D, Schilbach L, Vogeley K, Schneider K, Laird AR, Langner R, et al Parsing the neural correlates of moral cognition: ALE meta-analysis on morality, theory of mind, and empathy. Brain Struct Funct. 2012;217: 783–796. 22270812 10.1007/s00429-012-0380-y PMC3445793

[25] Mar RA. The neural bases of social cognition and story comprehension. Annu Rev Psychol. 2011;62: 103–134. 10.1146/annurev-psych-120709-145406 21126178

[26] Schurz M, Aichhorn M, Martin A, Perner J. Common brain areas engaged in false belief reasoning and visual perspective taking: a meta-analysis of functional brain imaging studies. Front Hum Neurosci. 2013;7: 712 10.3389/fnhum.2013.00712 24198773 PMC3814428

[27] Schurz M, Radua J, Aichhorn M, Richlan F, Perner J. Fractionating theory of mind: A meta-analysis of functional brain imaging studies. Neurosci Biobehav Rev. 2014;42: 9–34. 10.1016/j.neubiorev.2014.01.009 24486722

[28] Öngür D, Price JL. The organization of networks within the orbital and medial prefrontal cortex of rats, monkeys and humans. Cereb Cortex. 2000;10(3): 206–219. 10731217 10.1093/cercor/10.3.206

[29] Coricelli G, Critchley HD, Joffily M, O'Doherty JP, Sirigu A, Dolan R. Regret and its avoidance: a neuroimaging study of choice behavior. Nat Neurosci. 2005;8(9): 1255–1262. 16116457 10.1038/nn1514

[30] Small DM, Zatorre RJ, Dagher A, Evans AC, Jones-Gotman M. Changes in brain activity related to eating chocolate from pleasure to aversion. Brain. 2001;124(9): 1720–1733. 11522575 10.1093/brain/124.9.1720

[31] Pinel P, Piazza M, Le Bihan D, Dehaene S. Distributed and overlapping cerebral representations of number, size, and luminance during comparative judgments. Neuron. 2004;41: 983–993. 15046729 10.1016/s0896-6273(04)00107-2

[32] Zeki S, Stutters J. Functional specialization and generalization for grouping of stimuli based on colour and motion. Neuroimage. 2013;73: 156–166. 10.1016/j.neuroimage.2013.02.001 23415950 PMC3613798

[33] Cheadle SW, Zeki S. The role of parietal cortex in the formation of color and motion based concepts. Front Hum Neurosci. 2014;8: 535 10.3389/fnhum.2014.00535 25120447 PMC4112936

[34] Bird CM, Berens SC, Horner AJ, Franklin A. Categorical encoding of color in the brain. Proc Natl Acad Sci U S A. 2014;111: 4590–4595. 10.1073/pnas.1315275111 24591602 PMC3970503

[35] Bechara A, Tranel D, Damasio H, Adolphs R, Rockland C, Damasio AR. Double dissociation of conditioning and declarative knowledge relative to the amygdala and hippocampus in humans. Science. 1995;269: 1115–1118. 7652558 10.1126/science.7652558

[36] Heider ER, Oliver DC. The structure of the color space in naming and memory for two languages. Cognitive Psychology. 1972;3: 337–354.

[37] Mitchell JP. The false dichotomy between simulation and theory-theory: the argument's error. Trends Cogn Sci. 2005;9: 363–364; author reply 364. 16006173 10.1016/j.tics.2005.06.010

