# Supplementary material for: Differentiating Self-Projection from Simulation during Mentalizing: Evidence from fMRI
Source: PLoS One. 2015 Mar 25;10(3):e0121405. doi: 10.1371/journal.pone.0121405 (PMC4373917; doi:10.1371/journal.pone.0121405)
Supplement: S1 Table — (DOCX) [file pone.0121405.s001.docx]

**Supplementary Table S1.** Results of Linear Mixed Effects (LME) analysis of match between choices for self and other – only trials with perfect internal consistency for self and other choices.

|  |  |  |  |  |  |  |  |  |  |  |  |  |
| --- | --- | --- | --- | --- | --- | --- | --- | --- | --- | --- | --- | --- |
|  |  |  |  |  |  |  |  |  |  | Odd’s | Ratio |  |
|  | Df | AIC | BIC | logL | ChiSq |  | b | z | M | Lower Limit | Upper Limit |  |
|  |  |  |  |  |  |  |  |  |  |  |  |  |
| Baseline Model | 4 | 1668 | 1690 | -830 |  |  |  |  |  |  |  |  |
| +Target | 5 | 1661 | 1688 | -825 | 8.75** |  |  |  |  |  |  |  |
| +Color | 6 | 1663 | 1696 | -825 | 0.00 |  |  |  |  |  |  |  |
| +Inter. | 7 | 1651 | 1690 | -819 | 13.76*** | *Fixed effects:* |  |  |  |  |  |  |
|  |  |  |  |  |  | Intercept | 7.06 | 8.84*** | 1162 | 242.9 | 5561 |  |
|  |  |  |  |  |  | Target | -3.55 | -8.22*** | 0.12 | 0.09 | 0.50 |  |
|  |  |  |  |  |  | Color | -1.55 | -3.52*** | 0.03 | 0.01 | 0.06 |  |
|  |  |  |  |  |  | Inter. | 0.97 | 3.69*** | 2.64 | 1.58 | 4.43 |  |
|  |  |  |  |  |  |  |  |  |  |  |  |  |
|  |  |  |  |  |  |  |  |  |  |  |  |  |

Target… Varies if prediction was about similar versus dissimilar other. Color… Varies is prediction was made for similar versus dissimilar colors. Inter… Interaction between Target and Color. Df… Degrees of freedom, AIC Aikaike Information Criterion, BIC Bayesian Information Criterion, lokLik… log Likelihood, ChiSq… Chi Square, b… non-standardized b coefficient,* *p* < .05, * *p* < .01, * *p* < .001.
