# Supplementary material for: Differentiating Self-Projection from Simulation during Mentalizing: Evidence from fMRI
Source: PLoS One. 2015 Mar 25;10(3):e0121405. doi: 10.1371/journal.pone.0121405 (PMC4373917; doi:10.1371/journal.pone.0121405)
Supplement: S2 Table — Results are separately shown for conceptually similar and conceptually different colors. Voxel-level threshold of p<.001 and cluster extent p<.05 corrected. (DOCX) [file pone.0121405.s002.docx]

**Supplementary Table 2.** Results for self > control task. Results are separately shown for conceptually similar and conceptually different colors. Voxel-level threshold of *p* < .001 and cluster extent *p* < .05 corrected.

|  |  | MNI Coordinates | | |  |  |  |  |  | |
| --- | --- | --- | --- | --- | --- | --- | --- | --- | --- | --- |
| Label |  | x | y | z |  |  | *t* |  | Voxel Extent | |
|  | | | | | | | | | |  |
| **Similar Colors: Self > One Back** |  |  |  |  |  |  |  |  |  | |
| L IFG |  | -24 | 29 | -14 |  |  | 5.42 |  | 99 | |
| L paracentral |  | -12 | -32 | 52 |  |  | 5.23 |  | 139 | |
| L sup. frontal |  | -15 | 38 | 46 |  |  | 5.16 |  | - | |
| L post. cingulum |  | -3 | -49 | 25 |  |  | 5.11 |  | 69 | |
| R cuneus |  | 18 | -100 | 13 |  |  | 4.76 |  | 112 | |
| R lingual |  | 6 | -85 | -2 |  |  | 4.64 |  | - | |
|  |  |  |  |  |  |  |  |  |  | |
| **Different Colors: Self > One Back** |  |  |  |  |  |  |  |  |  | |
| L sup. frontal |  | -15 | 38 | 46 |  |  | 7.07 |  | 476 | |
| L sup. med. frontal |  | -9 | 59 | 31 |  |  | 5.08 |  | - | |
| L sup. med. frontal |  | -9 | 62 | 22 |  |  | 4.54 |  | - | |
| L sup. med. frontal |  | -9 | 59 | 1 |  |  | 4.10 |  | - | |
| L mid. frontal |  | -24 | 47 | 37 |  |  | 4.89 |  | - | |
| L med. orb. frontal |  | -9 | 56 | -5 |  |  | 3.86 |  | - | |
| R sup. med. frontal |  | 9 | 59 | 28 |  |  | 3.15 |  | - | |
| L mid. frontal |  | -33 | 17 | 49 |  |  | 5.34 |  | 151 | |
| L IFG triang. |  | -51 | 23 | 25 |  |  | 4.04 |  | - | |
| L precentral |  | -45 | 8 | 31 |  |  | 3.22 |  | - | |
| L IFG orbit. |  | -21 | 26 | -17 |  |  | 6.17 |  | 214 | |
| L IFG orbit. |  | -39 | 35 | -17 |  |  | 4.54 |  | - | |
| R rectus |  | 3 | 32 | -23 |  |  | 5.21 |  | - | |
| L rectus |  | -3 | 32 | -23 |  |  | 5.04 |  | - | |
| L IFG triang. |  | -48 | 38 | 13 |  |  | 3.94 |  | 50 | |
|  |  |  |  |  |  |  |  |  |  | |
| L postcentral |  | -36 | -22 | 46 |  |  | 4.72 |  | 105 | |
| L precentral |  | -39 | -19 | 58 |  |  | 4.22 |  | - | |
| L angular |  | -48 | -61 | 34 |  |  | 5.47 |  | 183 | |
| L angular |  | -48 | -70 | 37 |  |  | 5.32 |  | - | |
| L Precuneus |  | 0 | -64 | 31 |  |  | 6.87 |  | 377 | |
| L post. cingulum |  | -3 | -49 | 25 |  |  | 6.29 |  | - | |
| R cerebellum |  | 39 | -73 | -38 |  |  | 6.33 |  | 497 | |
| R cerebellum |  | 15 | -85 | -26 |  |  | 4.26 |  | - | |
| R cuneus |  | 15 | -100 | 13 |  |  | 6.01 |  | - | |
| R sup. occipital |  | 15 | -97 | 19 |  |  | 6.00 |  | - | |
| R fusiform |  | 27 | -76 | -11 |  |  | 4.56 |  | - | |
| R lingual |  | 15 | -82 | -11 |  |  | 4.40 |  | - | |
|  |  |  |  |  |  |  |  |  |  | |
